# Individual and combined associations of sarcopenia, osteoporosis and obesity with frailty in a multi-ethnic asian older adult population

**DOI:** 10.1186/s12877-023-04500-1

**Published:** 2023-12-05

**Authors:** Matthew Wong Yu Heng, Aurora W. D. Chan, Ryan E. K. Man, Eva K. Fenwick, Samuel T. H. Chew, Laura Tay, Ng Yee Sien, David Ng, Frederick H. Koh, Eu-Leong Yong, Preeti Gupta, Ecosse L. Lamoureux

**Affiliations:** 1 Singapore Eye Research Institute (SERI), The Academia, 20 College Road, Level 6, Singapore, 169856 Singapore; 2 University of Cambridge, Cambridge, England; 3 Duke-NUS Medical School, Singapore, Singapore; 4 Department of Geriatric Medicine, Changi General Hospital, Singapore, Singapore; 5 Department of Geriatric Medicine, Sengkang General Hospital, Sengkang, Singapore; 6 Rehabilitation Medicine, Division of Medicine, Singapore General Hospital, Singapore, Singapore; 7 Department of Nuclear Medicine, Singapore General Hospital, Singapore, Singapore; 8 Department of General Surgery, Sengkang General Hospital, Singapore, Singapore; 9 Department of Obstetrics & Gynecology, National University of Singapore, Singapore, Singapore; 10 The University of Melbourne, Melbourne, Australia

**Keywords:** Sarcopenia, Osteoporosis, Obesity, Frailty, Osteosarcopenia, Sarcopenic obesity, Osteo-obesity, Osteosarcopenic obesity, Older adults, Population-based

## Abstract

**Background:**

We explored the relationships between sarcopenia (SP), osteoporosis (OP), obesity (OB), (alone and in combination) with physical frailty (PF) in a multi-ethnic, population-based study of Asians aged ≥ 60 years.

**Methods:**

Participants were enrolled from the PopulatION HEalth and Eye Disease PRofile in Elderly Singaporeans Study (PIONEER) study. PF was defined using the modified Fried phenotype; SP using the Asian Working Group for Sarcopenia 2019; OP using bone mineral density scores; and OB using the fat mass index. Modified Poisson regression models investigated the associations between exposures and PF, and the relative excess rates of PF due to interactions (RERI) to determine synergistic or antagonistic interactions.

**Results:**

Of the 2643 participants, 54.8% was female; and 49.8%, 25.1%, 25.0% were Chinese, Indians, and Malays, respectively. 25%, 19.0% and 6.7% participants had OB only, SP only, and OP only, respectively. A total of 356 (17.5%), 151 (7.4%) and 97 (4.8%) had osteosarcopenia (OSP), sarcopenic obesity (SOB) and osteo-obesity (OOB), respectively; while 70 (3.5%) had all 3 morbid conditions (osteosarcopenic obesity, OSO). Both SP only and OB only were strongly associated with increased rates of PF (RR: 2.53, 95% CI: 1.95, 3.29; RR: 2.05, 95% CI: 1.58, 2.66 respectively); but not OP. Those with OSP, OOB and SOB were also associated with high risks of PF (RR: 2.82, 95% CI: 2.16, 3.68; RR: 2.34, 95% CI: 1.69, 3.23; and RR: 2.58, 95% CI: 1.95, 3.41, respectively) compared to robust individuals. Critically, individuals with OSO had the *highest* relative risk of having PF (RR: 3.06, CI: 2.28, 4.11). Only the sarcopenia-obesity interaction was significant, demonstrating *negative synergism (antagonism)*. The concurrent presence of SP and OB was associated with a 100% lower rate of PF compared to the sum of the relatively rates of SP only and OB only.

**Conclusion:**

The prevalence of SP, OB and OP, alone and combined, is substantial in older Asians and their early identification is needed to mitigate the risk of frailty. OB may interact with SP in an antagonistic manner to moderate rates of frailty. Further longitudinal studies are needed to address causality and mechanistic underpinnings our findings.

## Introduction

Singapore has one of the fastest ageing populations worldwide, with an estimated 1 in 4 Singaporeans projected to be aged ≥ 65 years in the next two decades [1]. This ageing phenomenon is expected to lead to an upsurge in age-related disorders, such as physical frailty (referred to as frailty henceforth), described as a “clinically recognizable state of increased vulnerability resulting from aging-associated decline in reserve and function across multiple physiological systems” [2]. The wide ranging impact of frailty on health outcomes, including diminished quality of life (QoL) and increased morbidity and mortality, imposes a significant personal and economic burden on patients, caregivers, and society [3]. As such, research is increasingly focusing on eliciting the risk factors associated with frailty to allow for its early detection and intervention.

Three age-related conditions have been postulated as potential modifiable risk factors for frailty, namely sarcopenia (SP), osteoporosis (OP), and obesity (OB) [4, 5]. SP refers to the age-associated loss of muscle mass, accompanied by low muscle strength and/or low physical performance [6]. Like frailty, it is classified as a age-related syndrome and is associated with several adverse health outcomes. While there is overlap between the two conditions, [7]. SP is believed to be one of the main drivers of frailty, with some considering it as a precursor state or a clinical manifestation [8]. SP is often accompanied by OP, the systemic loss of bone mineral and micro-architectural deterioration of bone tissue, and/or OB, a state of excess storage of body fat resulting from a chronic imbalance between energy intake and expenditure. Similar to SP, both OP and OB have been suggested to contribute to the development of frailty [5, 9]. While previously thought to be unrelated, evidence now suggests that these three conditions share common underlying genetic, environmental and mechanistic pathways [10].

Critically, several terms have been coined to reflect the co-presence of these conditions, such as osteo-sarcopenia (OSP), sarcopenic obesity (SOB), and osteo-sarcopenic obesity (OSO) [11], with high reported prevalence rates [12]. While studies have shown that the risks of frailty are significantly higher in those with OSP compared to SP or OP alone, [13, 14] data are currently missing in older Singaporean populations, a crucial knowledge gap given the ethnic variation in body composition, risk factor profile and disease outcomes in Asians compared to Caucasians [15]. Moreover, to date, our knowledge of the relationships between the concurrent presence of the 3 exposures of interest and frailty is incomplete [16, 17], with no study having evaluated the associations between osteo-obesity (OOB) and frailty. Finally, interactions between the 3 exposures with regards to frailty as an outcome have not been investigated. Importantly, whether a synergistic relationship exists between SP, OP and OB with the risk of frailty, i.e., whether their concurrent presence leads to an increased risk *above and beyond the risks associated with the sum of the component parts,* remains to be clearly established. Such data are crucial in further shaping clinical practice guidelines and informing resource allocation [16].

Against this background, this study explored the associations between SP, OP, and OB, individually and in combination, with frailty in an ethnically diverse, and older Asian population in the The PopulatION Health and Eye Disease Profile in Elderly Singaporeans Study (PIONEER). Interactions and possible synergisms/antagonisms between the exposures were investigated as well. We hypothesize that these three conditions (alone or combined) are independently associated with an increased likelihood of frailty, although the concurrent presence of these conditions will show greater odds of frailty compared to each condition in isolation.

## Methods

### Study population and design

PIONEER is a geographically-representative population-based study (2017–2022) investigating the clinical, biological, anthropomorphic, and psychosocial phenotypes of Chinese, Malay, and Indian Singaporeans aged ≥ 60 years. Participants were selected using an age, gender, and ethnicity-stratified framework based on the 2016 Singaporean population census residing across Singapore. PIONEER aims to better understand the epidemiology, burden, and complex mechanisms associated with age-related sensory deterioration. A detailed methodology is reported elsewhere [18]. Briefly, study invitation letters were sent out to 6,377 individuals selected using an age-, gender-, and ethnicity- stratified sampling framework from a national database. These individuals were followed up by study recruitment officers in a home visit to ascertain eligibility and agreement to participate. Of the 6,377 invited, 1,015 (15.9%) were classified as ‘uncontactable’ because of invalid address(s), were unresponsive to ≥ 3 home visit attempts, and/or living in residences that were inaccessible because of security restrictions. In addition, 648 (10.2%) individuals were excluded because they were incarcerated, were residing in nursing homes or outside Singapore, or were deceased; while a further 994 (15.6%) were deemed ineligible because they were terminally ill, bedridden or otherwise unable to give informed consent due to severe cognitive or hearing impairment or muteness. Of the remaining 3,720 (69.4%) eligible individuals, 2,643 (71.05%) took part in the study, 1,054 (28.33%) refused, and 23 (0.62%) were undecided. Reasons for refusal included lack of interest (*n* = 895, 84.9%) or time needed to participate in the study (*n* = 159, 15.1%). Compared to participants (*n* = 2,644), non-participants (*n* = 1,054) were older (*p* < 0.001), more likely to be female (*p* < 0.001), and Chinese (*p* < 0.001). Participants ranged from 60 to 100 years of age. About 54.8% of the sample was female, and 49.8%, 25.1%, 25.0% were Chinese, Indians, and Malays, respectively. PIONEER’s final respone rate was 71.5%.

The study was conducted at the research clinic of the Singapore Eye Research Institute. All study procedures were approved by the SingHealth Centralized Institutional Review Board (CIRB, Reference #2016/3089) and its protocol adheres to the principles of the Declaration of Helsinki. Written informed consent was obtained from all participants in either Chinese, Malay, Tamil or English.

#### Assessment and definition of frailty

Frailty was defined as presence of ≥ 3 conditions (Body mass index [BMI] < 18.5 kg/m^2^, low gait speed (< 1.0 m/s), low grip strength (men < 28 kg, women < 18 kg), exhaustion (score of < 10 for three questions from the vitality domain of the 12-item Short-form survey [SF-12]), low physical activity (gender-specific lowest quintile of total self-reported duration spent carrying out moderate and vigorous activity), modified according to the Fried phenotype [19]. Pre-frailty was defined as having 1 or 2 of these characteristics, and not frail (robust) as having none. A brief description of each measure is as follows:

1. BMI: Participants had their height and weight measured using a wall-mounted adjustable measuring scale and a calibrated digital scientific weight scale, respectively, and these data were used to calculate the individual’s BMI (weight in kg/height in m^2^);
2. Grip strength was determined using the handgrip strength test, and a digital hand dynamometer (JAMAR Plus +). Each participant’s dominant hand grip strength (kg) was measured three times in a seated position with elbows flexed at 90º. The mean grip strength score was recorded and utilized in analyses;
3. Gait speed was determined by a habitual gait speed test, with participants walking 4 m (15ft) at their usual speed, and timing (recorded in seconds) was stopped when the first foot completely crossed the 4 m mark;
4. Exhaustion was quantified with 3 questions taken from the vitality domain of the 12- item Short form survey (SF-12) used in the Medical Outcomes Study [20]: “Did you feel worn out?” “Did you feel tired?” “Did you have a lot of energy?” The questions were rated on a five-point Likert scale ranging from 0 (‘All of the time’) to 5 (‘None of the time’). The scores were summed, with a score of < 10 denoting exhaustion.
5. PA was assessed based on self-reported time (in hours) spent doing light (e.g., office work, driving a car, strolling), and moderate-vigorous activities (e.g., gardening, brisk walking, dancing, jogging).

#### Assessment and definition of SP, OP, OB, SOB, OSP, OOB, and OSO

Dual Energy X-Ray Absorptiometry (DXA; Discovery-W. Hologic Inc. Bedford-MA) was used to measure whole and regional body compositions, including fat and muscle mass, and bone mineral density (BMD). The DXA was performed at the research clinic of SERI by an Allied Health Professions Council accredited radiographer to ensure accuracy in positioning and delineation of bone map and region of interest.

Based on the Asian Working Group for Sarcopenia (2019) recommended cut-offs, SP was defined as having low appendicular muscle mass (men < 7 kg/m^2^, women < 5.4 kg/m^2^) in the presence of either low grip strength (men < 28 kg, women < 18 kg) or low gait speed (< 1 m/s) [6].

*BMD* was classified according to the World Health Organisation criteria for this age group, based on DXA measured T-scores of the lumbar spine, and/or femoral neck, and/or total hip as follows: normal (T-score > -1 SD), osteopenia (-1 ≥ T-score > -2.5 SD), and osteoporosis (T-score ≤ -2.5SD) [21]. Therefore, OP was defined a low BMD (T-score, ≤  − 2.5) in any of the following sites: hip, femoral, neck, and lumbar spine.

*Fat Mass Index (FMI)* was calculated by dividing the DXA-assessed individual’s fat mass (kg), by height (m) squared. A recent study conducted by Pang and associates in Singapore found FMI as the most preferred measure for obesity. Thus, we defined OB as FMI > 7.63 kg/m^2^ for men and > 9.93 kg/m^2^ for women, based on the sex-specific upper two quintiles of the Pang et al. study population [22].

SOB was considered present when SP and OB were both evident, OSP when OP and SP were encountered, osteo-obesity (OOB) for the concurrent presence of OP and OB, and OSO, when all 3 (OB, OP and SP) were present simultaneously.

#### Definition of covariates

In-house questionnaires were used to determine lifestyle factors such as smoking and alcohol habits, along with the patient’s medical history, including past occurrences of strokes, ischemic heart disease (IHD) and other systemic diseases. Venepuncture was conducted by a trained phlebotomist and 27 ml of non-fasting venous blood was used to perform various biochemistry tests. Blood and urine samples were processed and analysed at Quest Laboratories Pte Ltd (Singapore) for measurement on the same day. Diabetes was defined as haemoglobin A1c (HbA1c) > 6.5%, random blood glucose ≥ 11.1 mmol/L, use of diabetic medication, or self- reported diabetes. Chronic Kidney disease was defined as estimated glomerular filtration rate (eGFR) < 60 mL/min/1.73m^2^, based on the US National Kidney Foundation Kidney Disease Outcome (KDOQI) Working Group definition [23]. Hyperlipidemia was defined as high levels of total cholesterol (≥ 6.2 mmol/L) and/or self-reported use of lipid-lowering medications. IHD and stroke was defined as self-reported history of myocardial infarction or angina, and stroke, respectively, similar to previous epidemiologic studies conducted by our group. For blood pressure (BP), a digital automatic BP monitor was used with the participant seated and after 5 min of rest. Hypertension was classified as Systolic BP (SBP) ≥ 140 mmHg, diastolic BP (DBP) ≥ 90 mmHg, physician diagnosis, use of BP medication and/or self-reported hypertension. Cognitive impairment was defined based on 6-CIT score ≥ 8 or total montreal cognitive assessment (MOCA) score < 19 for individuals with education lower or equal to primary, < 22 for individuals with secondary or A levels and < 24 for individuals with tertiary education.

#### Statistical analyses

Characteristics of the study population were examined using counts and proportions for categorical variables. To determine the univariate association between sociodemographic, systemic and clinical variables and frailty status, chi-square tests were used. The relationship of OP, SP and OB, with frailty, was quantified using relative rates (RR) as determined by modified Poisson regression models. We additionally adjusted for age, gender, smoking status, alcohol status, presence of diabetes, hypertension, ischemic heart disease, stroke, chronic kidney disease, and cognitive impairment as confounders. Interactions between exposures were also calculated using the modified Poisson regression model. Once interactions were established, we evaluated the presence of synergistic or antagonistic effects using relative excess rates of frailty due to interactions (RERI). This technique was calculated according to a formula proposed by Katsoulis et and colleagues [24]. In particular, the three-way RERI (RERI_3_), and two-way RERI (RERI_2_) stratified by the third factor were generated. The interaction is super-additive (synergistic) when RERI_3_ or RERI_2_ is positive, while the interaction is sub-additive (antagonistic) when RERI_3_ or RERI_2_ is negative. The delta method was used to construct the 95% CI for RERI. If the 95% CI of the corresponding RERI does not include 0, interaction is present, and vice versa.

All statistical evaluations were made assuming a 2-sided test at the 5% level of significance. Statistical analyses were conducted using STATA version 17.0.

## Results

Of the 2643 participants, 2 aged < 60 years, 5 of ethnicities other than Chinese, Malays and Indians, as well as 422 with missing frailty status were excluded. As such, the remaining 2214 individuals (median [IQR] age was 72 [66–80] years) were included in the analyses, of which 1175 (53.1%) were female and 1118 (50.5%) were of Chinese ethnicity (Table 1). About one-quarter of our participants had OB only (24.4%), 19.0% had SP only, and 6.7% OP only. A total of 356 (17.5%), 151 (7.4%) and 97 (4.8%) had OSP, SOB and OOB, respectively; while 70 (3.5%) had all three morbid conditions under evaluation. Lastly, 1100 (49.7%) of participants were frail (Table 1). There were significant differences between frail and non-frail (robust alone or robust + prefrail) individuals, with frail individuals being older; of female gender; non-Chinese ethnicity; not currently consuming alcohol; and having comorbidities including, diabetes, hypertension, stroke, IHD, CKD, cognitive impairment, osteoporosis, sarcopenia and obesity (all *P* < 0.05; Table 1).

**Table 1:** Demographic, systemic, and socioeconomic characteristics of participants in the PIONEER study, stratified by frailty status (*N* = 2214)

	***n*** (%)			***P*-value**
	**Overall (*N* = 2214)**	**Not frail (*N* = 1114)**	**Frail (*N* = 1100)**	
Age group				< 0.001
60–69	898 (40.1)	592 (53.1)	306 (27.8)	
70–79	737 (33.3)	365 (32.8)	372 (33.8)	
≥ 80	579 (26.2)	157 (14.1)	422 (38.4)	
Gender				0.008
Male	1039 (46.9)	554 (49.7)	485 (44.1)	
Female	1175 (53.1)	560 (50.3)	615 (55.9)	
Race				< 0.001
Chinese	1118 (50.5)	660 (59.3)	458 (41.6)	
Malay	570 (25.8)	241 (21.6)	329 (29.9)	
Indian	526 (23.8)	213 (19.1)	313 (28.5)	
Diabetes	725 (36.3)	300 (29.4)	425 (43.5)	< 0.001
Hypertension	1877 (85.1)	905 (81.4)	972 (88.8)	< 0.001
IHD	353 (17.4)	140 (13.2)	213 (22.0)	< 0.001
Stroke	90 (4.3)	35 (3.3)	55 (5.5)	0.015
CKD	408 (20.4)	131 (12.7)	277 (28.6)	< 0.001
Cognitive impairment	297 (13.7)	55 (5.1)	242 (22.4)	< 0.001
OP	664 (32.3)	289 (27.3)	375 (37.5)	< 0.001
SP	965 (46.9)	372 (34.7)	593 (60.0)	< 0.001
OB (high FMI)	815 (40.0)	375 (35.7)	440 (44.5)	< 0.001
OSO triad				< 0.001
None	340 (16.7)	278 (26.6)	62 (6.3)	
OP only	135 (6.7)	96 (9.2)	39 (4.0)	
SP only	386 (19.0)	169 (16.2)	217 (22.0)	
OB only	496 (24.4)	260 (24.9)	236 (24.0)	
OP and SP only	356 (17.5)	129 (12.3)	227 (23.1)	
OP and OB only	97 (4.8)	41 (3.9)	56 (5.7)	
SP and OB only	151 (7.4)	55 (5.3)	96 (9.8)	
OSO	70 (3.5)	18 (1.7)	52 (5.3)	
Smoking status				0.311
Never smoked or past smoker	1990 (90.9)	995 (90.3)	995 (91.5)	
Current smoker	199 (9.1)	107 (9.7)	92 (8.5)	
Alcohol status				< 0.001
Never drank or past drinker	1901 (86.8)	904 (82.0)	997 (91.7)	
Current drinker	288 (13.2)	198 (18.0)	90 (8.3)	

Diabetes: HbA1c > 6.5%, random blood glucose ≥ 11.1 mmol/L, use of diabetic medication or self-reported; Hypertension: Systolic BP ≥ 140 mmHg, diastolic BP ≥ 90 mmHg, physician diagnosis, use of BP medication and/or self-report; Chronic kidney disease (CKD): Estimated glomerular filtration rate (eGFR) < 60 mL/min/1.73m2, based on the US National Kidney Foundation Kidney Disease Outcome Quality Initiative (KDOQI) Working Group definition. eGFR is estimated from the serum creatinine concentration (eGFR) using the CKD Epidemiology Collaboration (CKD-EPI) equation; Cognitive impairment: 6-CIT score ≥ 8 or total montreal cognitive assessment (MOCA) score < 19 for individuals with education lower or equal to primary, < 22 for individuals with secondary or A levels and < 24 for individuals with tertiary education; Osteoporosis: Presence of condition based on WHO classification from spine or hip DEXA scan; Sarcopenia: Low muscle mass and low grip strength or low gait speed; Obesity (High FMI): Gender-specific two upper quintile of FMI (> 8.51 kg/m2 for males and > 11.63 kg/m2 for females); Frail: Presence of ≥ 3 conditions (shrinkage, low gait speed, low grip strength, exhaustion, low moderate to vigourous physicial activity); Low grip grip strength: Average grip strength < 28 kg for males and < 18 kg for females; Low gait speed: gait speed < 1.0 m/s; Shrinkage: BMI < 18.5; Low moderate to physical activity: Gender-specific lowest quintile of total weekly moderate to vigorous physical activity (< 2 h for both males and females)

Missing diabetes (*n* = 217); hypertension (*n* = 7); IHD (*n* = 184); stroke (*n* = 136); CKD (*n* = 215); cognitive impairment (*n* = 46); osteoporosis (*n* = 155); sarcopenia (*n* = 155); obesity (*n* = 175); OSO triad (*n* = 183); smoking status (*n* = 25); and alcohol frequency (*n* = 25)

*Abbreviations:* *IHD* Ischemic heart disease, *CKD* Chronic kidney disease, *FMI* Fat mass index, *OSO* Osteoporosis-sarcopenia-obesity

Table 2 has 3 sections. In Sect. 1, we show the relative rate (RR) of OP, SP, and OB individually, and in combination with regards to frailty, adjusted for traditional confounders. Both SP only and OB only were strongly associated with increased RR of frailty (RR: 2.53, 95% CI: 1.95, 3.29; RR: 2.05, 95% CI: 1.58, 2.66 respectively); but not OP only (RR = 1.24, 95% CI: 0.83, 1.87). Those with OSP had a higher RR of frailty (RR: 2.82, 95% CI: 2.16, 3.68) compared to individuals with none of the three conditions. Additionally, compared to robust individuals, the presence of both OOB and SOB were also associated with higher RR of having frailty (RR: 2.34, 95% CI: 1.69, 3.23; and RR: 2.58, 95% CI: 1.95, 3.41, respectively). Critically, individuals with OSO (all 3 exposures combined) had the highest RR of having frailty (RR: 3.06, CI: 2.28, 4.11) when compared to persons with none of the three conditions.

**Table 2:** Estimated rate ratios of OP, SP and OB and of their product terms from modified Poisson regression, along with indexes of additive interaction between OP, SP and OB

**Adjusted Relative rates (RR) for each exposure group from our modified Poisson regression**		
**Exposure**	**Rate ratio (RR), 95% CI**	
None	Reference	
OP only	1.24 (0.83, 1.87)	
SP only	2.53 (1.95, 3.29)	
OB (high FMI) only	2.05 (1.58, 2.66)	
Both OP and SP only (OSP)	2.82 (2.16, 3.68)	
Both OP and OB only (OOB)	2.34 (1.69, 3.23)	
Both SP and OB only (SOB)	2.58 (1.95, 3.41)	
OP, SP and OB (OSO)	3.06 (2.28, 4.11)	
**Interactions between risk factors**		
**Risk factors and their interactions**	**Rate ratio (RR), 95% CI**	***P*-value**
OP-SP interaction	0.90 (0.58, 1.37)	0.612
OP-OB interaction	0.92 (0.58, 1.46)	0.718
SP-OB interaction	0.50 (0.37, 0.67)	< 0.001
OP-SP-OB interaction	1.16 (0.69, 1.96)	0.569
**Relative excess rate due to interaction (RERI)**		
	**RERI, 95% CI**	
RERI2 (OP, SP | no OB)^a^	0.04 (-0.55, 0.64)	
RERI2 (OP, OB | no SP)^a^	0.05 (-0.65, 0.74)	
**RERI2 (SP, OB | no OP)**^a^	**-1.00 (-1.67, -0.34)**	
RERI2 (OP, SP | OB)^b^	0.10 (-0.28, 0.48)	
RERI2 (OP, OB | SP)^b^	0.08 (-0.19, 0.35)	
RERI2 (SP, OB | OP)^b^	-0.68 (-1.49, 0.12)	
RERI3 (OP, OB, SP)^c^	0.16 (-0.83, 1.14)	

The modified Poisson regression model was adjusted for age, gender, race, diabetes, hypertension, IHD, stroke, CKD, cognitive impairment, smoking status and alcohol status

^a^RERI_2_(X1, X2 | X3 = 0) = RR_X1+X2+X3+_  − RR_X1+X2−X3-_ − RR_X1−X2+X3-_ + RR_X1−X2−X3-_

^b^RERI_2_(X1, X2 | X3 = 1) = (RR_X1+X2+X3+_  − RR_X1+X2−X3+_  − RR_X1−X2+X3+_  + RR_X1−X2−X3+)_ / RR_X1−X2−X3+_

^c^RERI_3_(X1, X2, X3) = RR_X1+X2+X3+_  − RR_X1+X2+X3−_  − RR_X1+X2−X3+_  − RR_X1-X2+X3+_  + RR_X1+x2-X3-_ + RR_X1-X2+X3-_ + RR_X1-X2-X3+_—RR_X1-X2-X3-_

In Sect. 2, we evaluated the interactions between OP, SP and OB. Only the sarcopenia only-obesity only interaction was significant (*p* < 0.001; Table 2).

In Sect. 3, we evaluated the excess RR of having frailty (Relative excess rate due to interaction, RERI) due to potential interactions among the three conditions, if any. Interestingly, we found that the concurrent presence of SP and OB (SOB) resulted in a 100% (RERI: -1.00, 95% CI: -1.67, -0.34) *decrease in the expected cumulative* rates of frailty estimated from the individual reported RRs of the two exposures.

## Discussion

In our large, cross-sectional, population-based study of older Singaporeans, we investigated the relative rates of having SP, OB and OP, individually or in combination, on frailty. We found that all combinations, except OP only, were significantly associated with greater likelihood of frailty. Having all three exposures concurrently (OSO) was associated with the highest risk of frailty. Interestingly, the concurrent presence of SP and OB (SOB) appeared to moderate the rates of frailty compared to what would be expected based on the individual reported rate ratio profile of SP and OB, thus demonstrating negative synergism (antagonism). Our findings suggest that SP and OB may play an important role in the pathogenesis of frailty and could be utilized as early indicators/biomarkers of this debilitating health condition. These results also highlight the importance of regular screening and early diagnosis and intervention for both SP and OB as part of clinical frailty management. Moreover our findings demonstrates that the prevalence of 2 or more of the OSO triad is common. This suggests that a multimodal intervention program that incorporates nutrition, strength, endurance and balance training may be beneficial and cost-effective for the physically frail. Longitudinal studies with multi-omics analyses are needed to validate the cause-effect and potential antagonistic nature, as well as the mechanisms underpinning the impact these exposures on incident frailty.

Our finding that SP alone is associated with frailty is supported by several other studies [8, 25]. In contrast, the relationships between OB and OP with frailty are not as consistent. For instance, OB was found to be associated with increased frailty risk in multiple studies in America (The woman’s health and ageing study), [26] France (the Gazel cohort), [27] China [28] and Europe (NHANES cohort), in contrast with others that demonstrate no such associations, such as the Concord Health and Ageing in Men Project [29]. Our study corroborates with the former studies and demonstrates a strong association between OB and frailty. Likewise for OP, evidence is equivocal with some studies suggesting a significant positive association with frailty, [30, 31] while others have observed no such relationship [32]. Here, our study demonstrates a lack of association between OP and frailty; and further studies are needed to verify these results. However, these differences in literature may be due to the difference in mechanism or pathophysiology of how OP and SP results in frailty, the distinct study population characteristics, the different number of years of follow-up and how frailty was assessed and defined.

Some studies have also investigated these exposures in combination, with the majority demonstrating an exacerbated odds of frailty in comparison to the presence of individual exposures only. For example, a cross sectional study in Chinese community-dwelling adults [13] and the Hertfordshire cross-sectional study [4] found that the likelihood of frailty was higher in the presence of OSP than in the presence of SP or OP alone. This corroborates with our results. To our knowledge, there have been no studies investigating the relationship of osteoporotic-obesity (OOB) to frailty. Only 2 studies have investigated the association between the concurrent presence of all 3 conditions (OSO) with frailty, with both studies reporting high odds ratios [16, 17]. Similarly, our study found that OSO was associated with the highest rates of frailty out of all our exposures. For SOB, most literature suggests that it carries an exacerbated risk for frailty compared to SP and OB alone [33]. For instance, the recent WCHAT study in China demonstrated an increased odds of frailty for SOB compared to SP or OB alone [34]. Interestingly, a recent study conversely found that the odds of frailty for SOB were lower than the risks for SP *alone*, concluding that obesity accompanying sarcopenia might thus lower the risks of developing frailty [35]. Our results clearly show that SOB is associated with a heightened risk of frailty, higher than the relative rates of SP and OB alone. We found however, that there was *negative* synergism between SP and OB, where the presence of these two conditions resulted in a statistically significant reduction in the rates of having frailty compared to their expected cumulative individual contributions.

Having investigated interactions between our exposures, we found the only significant interaction between SP and OB, which demonstrated negative synergism. The lack of interaction between other exposures such as SP and OP is unexpected, given the well-established cross talk between muscle and bone [11, 36]. Moreover, several tissue specific factors released by muscle have been shown to modulate bone such as insulin like-growth factor 2 (IGF2) and fibroblast growth factor 2, [36] while both osteoblasts and osteocytes have been shown to produce specific molecules such as prostaglandin E2 and osteocalcin that may impact muscle cells. For the interaction between SP and OB, we hypothesize that this negative synergism exists as excess adiposity later in life may actually serve as a form of energy reserve. Having excess adipose tissue may be protective against the presence of any protein-energy malnutrition in older adults, [37] and may provide other benefits such as an increase in cognitive function/reduction in cognitive decline, [38, 39] possibly contributing to reduced frailty [40]. We anticipate protein energy malnutrition is the most likely cause as it is a major risk factor for sarcopenia, as well as frailty [41]. Even in the original Fried phenotype for frailty, weight loss of more than 5% or 10 pounds was the first criterion in the assessment for frailty. In addition, a large UK Biobank study suggest that frailty was present in 92.1% of people diagnosed with malnutrition (undernutrition) [42].

Strengths of our study include the exclusive focus on older participants (≥ 60 years), an under researched section of our population, with a potential to offer novel insights into the world of an older individual, across three ethnic groups. Multivariate adjustment for a range of relevant confounders such as smoking and hypertension were conducted. Our comprehensive examination protocol, along with our usage of standardized measurements of clinical characteristics, such as the Fried Frailty Index, and our definition of Sarcopenia (AWGS 2019) make our results more widely applicable. Limitations of our study include the cross-sectional nature of our study, which preclude definitive conclusions about causality. Longitudinal follow-up of the participants is needed to determine a temporal association, and to address it we have already started the 4-year follow-up data for the PIONEER-2 study which will be able to shed light on these causal relations. There were also small numbers in each of the exposures, particularly concurrent exposures such as in OSO, which might have resulted in spurious findings. In addition, although, DXA is the current gold standard for measuring body parameters such as fat and appendicular lean mass (ALM), its assessment of fat and ALM may not be precise due to inclusion of non-contractile tissue such as water (both extracellular and intracellular), and other non-bone and non-fat soft tissues [43–45]. As such, DXA may overestimate fat mass in patients with edema or ascites, which is likely prevalent in older population [46]. This limitation may have led to an overestimation of the number of obese participants. Finally, for those participants with limited mobility (e.g., wheelchair bound), we initially offered the option to undergo study testing in a custom-designed mobile eye clinic (MEC), equipped with all the necessary equipment to undertake the study protocol. However, the MEC protocol was subsequently withdrawn after pilot testing revealed substantial technical and logistical difficulties (limited parking lots, lack of a washroom to accommodate the lengthy study protocol). This decision may have led to some selection bias, in that certain individuals who were frail may have been unable to participate in the PIONEER assessment. Nevertheless, participants with mobility issues and who agreed to the study were fetched tok and from the research clinic for assessments. Our data should thus be interpreted with caution.

In conclusion, we found that all combinations of SP, OP and OB, aside from OP alone, were significantly associated with greater likelihood of frailty. Interestingly, we showed a possible negative synergism between SP and OB with regards to frailty. These findings highlight the importance of screening for these exposures to aid early identification of frailty and amelioration of its development. Our findings also highlight that the prevalence of 2 or more of the OSO triad is common. This suggests that a multimodal intervention program that incorporates nutrition, strength, endurance and balance training may be beneficial and cost-effective for the physically frail. However, further studies are needed to verify the significance and strengths of these associations and interactions.

## Data Availability

All data generated or analysed during this study are included in this article.
